# Dry Eye Symptoms in Jazan University Lecturers During the COVID-19 Pandemic Using Ocular Surface Disease Index (OSDI)

**DOI:** 10.7759/cureus.49123

**Published:** 2023-11-20

**Authors:** Ismail Abuallut, Eman Hurissi, Ethar A Khawaji, Ghada Khormi, Rahaf Othathi, Fahad Y Azyabi, Abdulaziz Awlaqi, Mohammed Ghazi M Bakreen, Saleh Ghulaysi

**Affiliations:** 1 Ophthalmology, Jazan University, Jazan, SAU; 2 Medicine and Surgery, Jazan University, Jazan, SAU; 3 Emergency Medicine, Jazan University, Jazan, SAU; 4 College of Medicine, Jazan University, Jazan, SAU

**Keywords:** jazan, pandemic, covid-19, ded, osdi

## Abstract

Background

Dry eye disease (DED) is a disease of the ocular surface charac­terized by instability of the tear film, which causes ocular surface inflamma­tion and damage that leads to ocular symptoms, discomfort, and visual disturbance. Dry eye is a common ocular condition and a major reason for ophthalmologist visits. Compulsory e-learning has arisen in colleges and schools with the coronavirus disease 19 (COVID-19) pandemic as a tool for new teaching and learning. DED is an emerging threat to public health and is directly proportional to digital screen viewing length. DED diagnosis flowchart begins with history-taking of associated risk factors and a screening test by Ocular Surface Disease Index (OSDI). Therefore, we aim to assess the prevalence and the severity of DED among Jazan University lecturers and to identify the associated risk factors.

Methods

A total of 150 participants were recruited for this descriptive, observational study. Participants completed an online questionnaire that contained questions about sociodemographics, electronic devices they used, the average number of hours of use in a day as well as the distance and posture while reading, and factors that may influence visual symptoms such as the use of glasses, frequent changes in glasses prescription and DED symptoms.

Results

The results showed that the prevalence of DED was high, with 23% of participants having mild DED, 12% having moderate DED, and 29% having severe DED. DED was associated with a number of sociodemographic and clinical factors, including younger age, female gender, occupation as a lecturer, and use of digital devices.

Conclusion

This study highlights the need to develop strategies to prevent and control DED among high-risk groups, such as university lecturers. Future research should focus on identifying more effective ways to prevent DED and to improve the management of DED symptoms.

## Introduction

Multiple factors play roles in the development of dry eye disease (DED). It's a disease of the ocular surface characterized by instability of the tear film, which causes ocular surface inflammation and damage that leads to ocular symptoms, discomfort, and visual disturbance. New data suggests that neurosensory abnormalities are included in the definition of DED [1].

The main symptom of DED is dryness and a gritty feeling; in addition, the complaint includes searing or itching in the eyes, foreign body sensation, extra tearing, discomfort, redness of the eyes, and photophobia. [2]. In 2017, Stapleton et al. conducted a review of the available evidence on dry eye. The prevalence of DED ranges from 5 to 50 percent globally, according to epidemiological studies. The prevalence was higher in women and the elderly population. Moreover, DED is more prevalent in Asian countries compared to Western countries. It's associated with lower quality of life and reduced work productivity, particularly through vision loss and pain [3]. DED is dramatically increasing in Saudi Arabia, and it's a common ocular condition and a major reason for ophthalmologist visits. The age-adjusted DED prevalence in the Saudi Arabian population ranged from 7% to 32.1% [4,5]. DED has been associated with comorbidities, including musculoskeletal, neurological, ophthalmic, allergic, physical, dermatological, and atopic disorders [6]. 

In 2014, Paulsen et al. conducted a cohort study to investigate the risk factors for DED. The results of the study showed that significant associations were found with female sex, current contact lens use, allergies, arthritis, thyroid disease, antihistamine use, and steroid use [7]. Recent studies published to investigate the prevalence of DED during the COVID-19 pandemic and the effect of e-learning on the development of DED In 2022, Wejdan Alnahdi et al. conducted a study to measure the prevalence of DED and its association with screen time in the pediatric population during the COVID-19 pandemic using the Ocular Surface Disease Index (OSDI) questionnaire. The results of the study showed an increase in the symptoms of DED during the COVID-19 pandemic due to prolonged screen time [8]. Another study conducted by Santa-Cruz-Pavlovich et al. in 2023 aimed to assess the difference in the incidence of DED since the onset of online courses started using OSDI. The results of this study showed that since the online courses started, university students have complained of severe DED symptoms [9]. Moreover, in 2023, Lulla et al. conducted a study to investigate the changes in the prevalence of DED among medical students during COVID-19 and compare it to pre-pandemic periods using a modified OSDI questionnaire. The prevalence of DED was 41.5 during the pre-pandemic period and 55.19 during the pandemic period [10]. Therefore, we aim to assess the prevalence and severity of DED among Jazan University lecturers and identify the associated risk factors.

## Materials and methods

A cross-sectional descriptive study was conducted at Jazan University, Jizan City, Kingdom of Saudi Arabia, in 2021. The study applied to all lecturers at Jazan University who used e-learning during the COVID-19 pandemic, including males, females, Saudis, and non-Saudis. The research team translated previously OSDI-used English into Arabic. The questionnaire is divided into three parts. The first part included questions on sociodemographic characteristics. In the second part, participants were asked questions about the electronic devices they used, the average number of hours of use in a day, as well as the distance and posture while reading. Additional information on factors that might have influenced visual symptoms was collected, such as the use of glasses, frequent changes in glasses prescription, and the use of smartphones at bedtime with lights switched off. The third part assessed DED symptoms using a series of questions about visual symptoms such as headaches, blurred vision, fatigue, etc. Each question had five answer choices from zero to four (0 = never, 1 = mild, 2 = moderate, 3 = severe, 4 = very severe). Data were collected in an Excel sheet (Microsoft, Redmond, Washington), double-checked, coded, and transferred to statistical software for analysis (SPSS version 25; IBM Inc., Armonk, New York). For descriptive statistics, the results were presented as frequencies and percentages (demographic and eye-related variables), mean and standard deviations (OSDI scores), and figures (OSDI severity). The data were then subjected to a normality test, which revealed a non-normal distribution (Shapiro-Wilk test, p<0.05). Accordingly, the non-parametric Mann-Whitney U test was used to determine the differences in OSDI scores between the different categories. A p-value of 0.05 was considered significant. Approval for this study was obtained from the Ethical Committee of Jazan University (approval No. REC-43/02/006). Before data collection, patients' permission was obtained. The participant's data are stored with strict confidentiality in a computerized registry.

## Results

A total of 150 faculty members completed the survey. About two-thirds (70.0%, n=145) were non-medical, while more than half (56.0%, n=84) were ≤40 years old. The majority of the participants were female (76.7%, n=115), lecturers (86.0%, n=129), non-smokers (95.3%, n=143), and had no systemic disease (80.7%, n=121). More details are presented in Table 1.

**Table 1:** Characteristics of the study population

	Frequency	Percentage (%)
Specialty		
Medical	45	30.0
Non-medical	105	70.0
Age		
≤40 yrs	84	56.0
>40 yrs	66	44.0
Sex		
Male	35	23.3
Female	115	76.7
Faculty position		
Lecturer	129	86.0
Teaching assistant	21	14.0
Smoking		
Yes	7	4.7
No	143	95.3
Have systemic disease		
Yes	29	19.3
No	121	80.7

**Figure 1:**
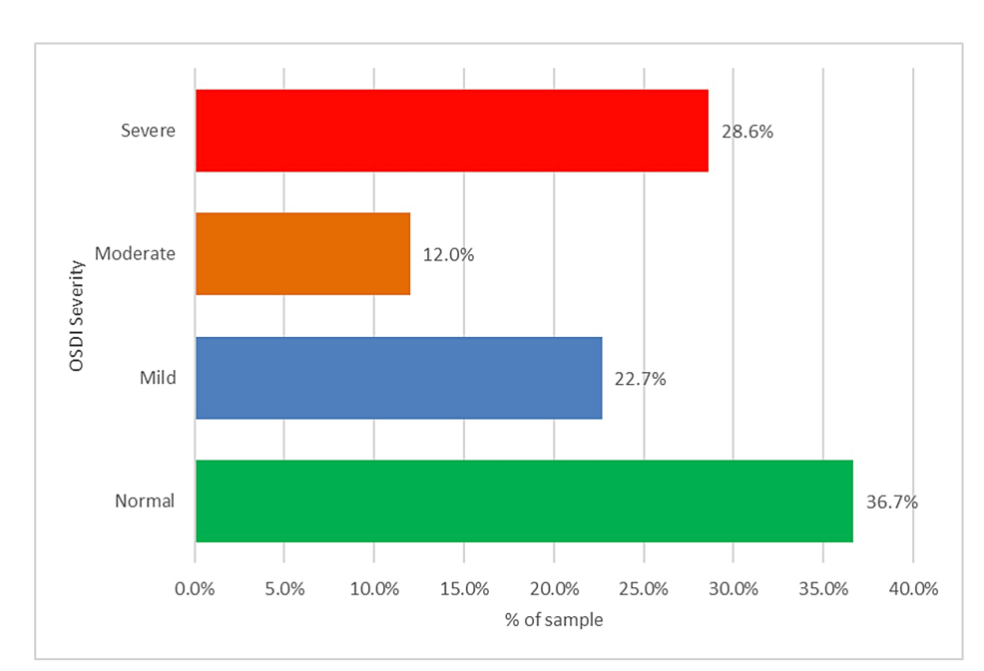
Distribution of OSDI severity degrees among the study population OSDI - Ocular Surface Disease Index

The degree of OSDI severity among the participants is shown in Figure 1. Over one-third (36.7%) of the participants were normal, (22.7%) had mild OSDI, 12% had moderate OSDI, and 28.6% had severe OSDI.

As shown in Table 2, about one-third (32.7%) of the participants used eye medications, 28.0% had previous ocular surgery, and 70.0% claimed that they felt eye fatigue (dryness). Fifty-eight (38.7%) participants used mobile phones for over six hours before COVID-19. However, this number increased substantially to 95 (63.3%) participants after COVID-19. Forty-one (27.3%) participants maintained a distance of 25 cm between the eye and mobile, while 109 (72.7%) maintained a higher distance. Most participants (76.7%) reported using their mobile before bedtime. Half of the participants (50%) wore eyeglasses, and 44.7% changed the size of their glasses after COVID-19.

**Table 2:** Responses of the participants to the eye-related variables

	Frequency	Percentage (%)
Using eye medication		
Yes	49	32.7
No	101	67.3
Had ocular surgery		
Yes	42	28.0
No	108	72.0
Feeling eye fatigue (dryness)		
Yes	105	70.0
No	45	30.0
Time spent using mobile before COVID-19		
≤6 hrs/day	92	61.3
>6 hrs/day	58	38.7
Time spent using mobile after COVID-19		
≤6 hrs/day	55	36.7
>6 hrs/day	95	63.3
Distance between eye and mobile		
25 cm	41	27.3
>25 cm	109	72.7
Using mobile before bedtime		
Yes	115	76.7
No	35	23.3
Wearing eyeglasses		
Yes	75	50.0
No	75	50.0
Changed the size of the glasses		
Yes	67	44.7
No	83	55.3

Subjects in the medical field showed higher OSDI scores (26.8±23.4) than those in the non-medical field (24.5±19.0) with (p=0.984). Younger subjects (≤40 years) reported higher OSDI scores than older subjects (26.5±21.4 compared to 23.4±19.0; p=0.374). In addition, teaching assistants, non-smokers, and subjects with systemic disease recorded higher OSDI scores than their counterparts but with (p>0.05). Which, as shown, indicated no significant difference. However, Female subjects reported significantly higher OSDI scores than males (27.4±21.2 compared to 17.7±15.4; p=0.016). More details are presented in Table 3.

**Table 3:** Differences in OSDI scores among participants according to the demographic variables OSDI - Ocular Surface Disease Index

Item	Response	OSDI score	
		Mean±SD	p-value
Specialty	Medical	26.8±23.4	0.984
	Non-medical	24.5±19.0	
Age	≤40 yrs	26.5±21.4	0.374
	>40 yrs	23.4±19.0	
Sex	Male	17.7±15.4	0.016
	Female	27.4±21.2	
Faculty position	Lecturer	24.9±20.2	0.814
	Teaching \ssistant	26.8±22.0	
Smoking	Yes	23.8±19.0	0.964
	No	25.2±20.5	
Have systemic disease	Yes	30.0±21.7	0.161
	No	24.0±19.9	

Subjects who used eye medications scored higher OSDI than their counterparts but with (28.9±21.2 compared to 23.3±19.8; p=0.089). Similarly, subjects with previous ocular surgery, who spent >6 hours/day using mobile before and after COVID-19, and who maintained a 25 cm distance between the eye and mobile, scored higher OSDI than their counterparts but with (p>0.0%) which lastly indicated no significant difference. However, subjects who felt eye fatigue, wore eyeglasses, and changed the size of their glasses recorded significantly higher OSDI scores than their counterparts (P< 0.05). On the other hand, subjects who used mobile before bedtime recorded lower OSDI scores than their counterparts (24.6±20.5 compared to 27.2±20.1) but with no significant difference (p=0.365).

**Table 4:** Differences in OSDI scores among participants according to the eye-related variables

Item	Response	OSDI score	
		Mean±SD	p-value
Using eye medication	Yes	28.9±21.2	0.089
	No	23.3±19.8	
Had ocular surgery	Yes	27.0±18.8	0.220
	No	24.4±21.0	
Time spent using mobile before COVID	≤6 hrs/day	25.1±19.3	0.773
	>6 hrs/day	25.3±22.1	
Time spent using mobile after COVID	≤6 hrs/day	23.6±18.5	0.690
	>6 hrs/day	26.1±21.4	
Distance between eye and mobile	25 cm	27.4±23.5	0.577
	>25 cm	24.3±19.1	
Feeling eye fatigue (dryness)	Yes	28.6±20.1	0.000
	No	17.1±18.9	
Wearing eyeglasses	Yes	31.1±21.2	0.000
	No	19.3±17.8	
Changed the size of your glasses	Yes	32.3±22.8	0.000
	No	19.4±16.1	
Using mobile before bedtime	Yes	24.6±20.5	0.365
	No	27.2±20.1	

## Discussion

During the COVID-19 pandemic, several countries, including Saudi Arabia, imposed measures to control the spread of the virus. One of these measures is lockdown. The educational system in schools and universities has been changed to online. Increased screen and electronic device exposure time can create a longer blinking interval, which can increase tear evaporation and lead to the development of DED [11]. In 2017, the prevalence of DED, according to Dry Eye Workshop (DEWS) II, ranged from 20% to 50% [3]. In our study, we aimed to assess the prevalence and severity of DED among Jazan University lecturers during the COVID-19 pandemic and identify the associated risk factors based on the OSDI questionnaire. The OSDI is a tool used to measure DED-related symptoms' severity and their related visual function impairment. OSDI has high sensitivity and specificity, reaching 60% and 79%, respectively [12].

In our study, 150 faculty members participated in filling out the OSDI form. According to the OSDI diagnostic criteria, over one-third (37% of the participants) had no DED symptoms. Nearly two-thirds (63%) of the participants experienced DED with various degrees of severity during the pandemic. Furthermore, we found that the degree of OSDI severity among the participants was 23% mild OSDI, 12% moderate OSDI, and 29% severe OSDI. High cases of DED during the pandemic have been reported in many studies. In 2022, a study conducted by Alnahdi et al. aimed to measure the prevalence of DED and its association with screen time in the pediatric population during the COVID-19 pandemic using the OSDI questionnaire. The results of the study showed that 76.1% of participants had symptomatic DED, while 23.9% had no DED. The authors found an increase in the symptoms of DED during the COVID-19 pandemic due to prolonged screen time exposure [8]. Another study conducted in 2022 by Garca-Ayuso et al. aimed to assess the prevalence of DED using the OSDI questionnaire among university students. The results of the study showed that the prevalence of symptomatic DED was 51.8% [11]. Moreover, in 2023, Lulla et al. conducted a study to investigate the changes in the prevalence of DED among medical students during the COVID-19 pandemic and compare it to pre-pandemic periods using a modified OSDI questionnaire. The prevalence of DED was 41.5 during the pre-pandemic period and 55.19 during the pandemic period [10]. Another study conducted by Pavlovich et al. in 2023 aimed to assess the difference in the incidence of DED since the onset of online courses using the OSDI questionnaire. The results of this study showed that since the online courses started, university students have complained of severe DED symptoms [9].

Globally, the prevalence of DED was higher in women and the elderly population [3]. However, our study showed a higher OSDI score in females and younger subjects, and our results are consistent with the results of the Tripathi et al. study that was conducted in 2022. The study aimed to determine the magnitude and severity of DED. The study results reported an increase in the prevalence of symptomatic DED from 25% to 41% in young participants [13].

A large number of cross-sectional studies have explained the association between digital screen time exposure and DED. A study conducted among 3549 office workers who use visual display terminals (VDTs) by Uchino et al. aimed to assess the prevalence of DED and its associated risk factors. The results of the study showed that the participants who used digital screens for more than four hours per day experienced severe symptoms of dry eye [14]. Moreover, Lulla et al.'s study revealed an increase in DED symptoms by 1.7 times during the pandemic period in students who spent more than five hours on a digital screen [10]. Also, Iqbal et al. Found an association between an increased risk of DED development and an increase in screen time for more than three hours [15]. These findings are consistent with our results that showed higher OSDI scores in subjects who spent more than six hours per day using the mobile before the pandemic (25.3 OSDI score) and after the pandemic (26.1 OSDI score). The OSDI score for the subjects who spent more than six hours/day using mobile was 25.1 before the pandemic and 23.6 after the pandemic. However, no significant difference has been shown in our study.

In our study, we found higher OSDI scores in the participants who had undergone previous ocular surgery. Ishrat et al. found similar results in a study aimed at investigating the incidence of DED after cataract surgeries. Fifty percent of the patients developed DED at one  month, and 9% developed DED at three months after surgery [16].

In addition, in 2012, Fraunfelder et al. conducted a review that explained the relationship between DED and the use of multiple systemic and topical ocular medications. The authors found that ocular medications, either systemic or topical, can cause DED through various mechanisms [17]. This result is consistent with our results, which found a higher OSDI score in participants who use ocular medications.

Moreover, we found a higher OSDI score in subjects who had a history of systemic diseases. In 2021, Yu et al. conducted a large multi-center randomized clinical trial on DED patients. The authors found that patients who have systemic diseases experienced more severe DED signs in comparison with patients without systemic diseases [18].

Furthermore, in our study, we found that higher OSDI scores were reported in subjects in the medical field, teaching assistants, and non-smokers. Regarding the association between DED and smoking, Tariq et al. conducted a meta-analysis, and the results indicate that smoking doesn't increase the risk for DED [19].

This study has limitations; there is possible self-selection bias as the survey was distributed online. However, to the best of our knowledge, this is the first study that analyzed the prevalence of DED using a standard tool such as OSDI among Jazan University lecturers during the COVID-19 pandemic. Further, this study highlighted the high prevalence of DED associated with e-learning and called attention to the importance of early diagnosis and management of DED, especially in the highest-risk group, to prevent some of the severe complications.

## Conclusions

In this study, we found a high prevalence of DED with various degrees of severity according to OSDI. Younger age, female gender, occupation as a lecturer, and use of digital devices were associated with DED. This study highlights the need to develop strategies to prevent and control DED among high-risk groups, such as university lecturers. Future research should focus on identifying more effective ways to prevent DED and to improve the management of DED symptoms.
